# A descriptive analysis of children seeking medical attention for problematic sexualized behavior

**DOI:** 10.3389/fpsyt.2023.1272789

**Published:** 2023-11-10

**Authors:** Kara Thompson, Sasha Svendsen

**Affiliations:** ^1^UMass Memorial Children’s Medical Center, Worcester, MA, United States; ^2^Department of Pediatrics, University of Massachusetts Chan Medical School, Worcester, MA, United States

**Keywords:** problematic sexualized behaviors, child abuse, sexual abuse, child protection, neglect

## Abstract

**Introduction:**

Problematic Sexualized Behavior (PSB) in children is an increasingly prevalent and often misunderstood issue that impacts the well-being of children. Quantifying the numbers of affected children is challenging due to inconsistencies in how these children are identified, as well as misperceptions surrounding the issue and lack of a coordinated community response.

**Methods:**

In this single-center retrospective case review, we attempt to report data on child demographics and case characteristics for children presenting to one Child Protection Program (CPP) medical specialty team for concern of PSB.

**Results:**

A total of 224 children were identified as having engaged in PSB during the study period. 110 (49%) of these children were referred to the CPP for medical evaluation and medically triaged by the team. The remaining 114 children (51%) were identified through the medical triage of the presenting cases as having engaged in PSB with the index children, but were not referred to CPP for medical care themselves. The majority of children who were referred (69%) were the recipient of the PSB, compared to being the displayer of the behavior (20%). Of the recipient cases, the child displaying PSB was also referred to the CPP only 14.5% of the time.

**Discussion:**

These results highlight that the number of children presenting for medical evaluation with concern of PSB is a significant underestimation of the prevalence of PSB in the community. This notable gap in identification of children engaging in PSB prevents service delivery for these children, including medical evaluation. The results also demonstrate that children displaying PSB were disproportionately missing from care and represent a specific area of missed opportunity for intervention and support by medical professionals.

## Introduction

Problematic Sexualized Behavior (PSB) in children is an increasingly prevalent and often misunderstood issue that impacts the well-being of children. There is no single sexual behavior that has been identified as pathognomonic for a sexual behavior problem, thus making recognition and understanding of PSB challenging (1). It is important to consider several characteristics about the behavior when distinguishing between typical vs. problematic sexual behavior, including the frequency of the behavior, the child’s developmental stage, and the level of harm involved (2). PSB is commonly defined as behaviors in children ages 12 years and younger that involve sexual body parts, are developmentally inappropriate, and may be harmful to themselves or others (2, 3).

Quantifying the number of affected children is challenging due to inconsistencies in how these children are identified (4). Estimates from Children’s Advocacy Centers (CACs) across the United States suggest that 20–25% of cases served by CACs involve youth acting out against another child (5). This number likely underestimates the total number of children impacted annually, as many are never brought to the attention of CACs or other community agencies. While evidence suggests that PSB occurs internationally, the available research is heavily concentrated in middle to high income countries, including the United States, United Kingdom, and Canada (6, 7).

The existing literature on PSB in children suggests that there may be risk factors associated with these behaviors. One common misperception is the assumption that all children displaying PSB have been sexually abused in the past. While sexual abuse has been shown to be a risk factor for displaying PSB, the prevalence of prior sexual abuse among PSB cases was found to be as low as 38% (2, 8, 9). Several other risk factors for PSB have been demonstrated to occur with significantly greater prevalence. For example, interpersonal violence was found in 68% of children presenting with PSB in the same study referenced above (9). A history of physical abuse was also identified in 47% of PSB cases (9). Other risk factors documented in the literature include high parental stress, neglect, and exposure to sexually explicit media (1, 8–13). A review by Elkovitch et al. (14) highlighted how the intersection of several risk factors plays a more impactful role in the development of PSB than any one risk factor (14). Research is needed to further characterize these risk factors and assess their impact on PSB.

Another misperception surrounding this topic is that PSB is comparable to sexual crimes committed by older adolescents and adults. Children who display PSB with other children are often labeled as “perpetrators,” and viewed through the criminal lens, or considered victims of sexual abuse, although neither may be true (5, 12). The Survey on Youth with Problematic Sexual Behaviors also found that 67.8% of professionals in child-serving roles perceived children who displayed PSB to be similar to adult sexual offenders (15). Recent literature demonstrates that criminalizing the behavior and using punitive responses with children displaying PSB does a disservice to these children, who are in fact no more likely to perpetrate sexual abuse as adults when provided appropriate therapeutic intervention (16). These misinformed perceptions negatively impact the treatment of children affected by PSB and their ability to access care.

Further compounding these challenges is the lack of a coordinated community response or national standard of care to address these cases and support families. Not uncommonly, due to state legal regulations, responding to concerns of PSB falls beyond the scope of the agencies typically charged with keeping children safe, such as child welfare and law enforcement (4, 12). As a result, families often do not know who to turn to for help. The Survey on Youth with Problematic Sexual Behaviors conducted by the National Children’s Alliance in 2020 found that among members of CAC’s nationwide, 35.3% of respondents reported that their communities do not have a structured or consistent response to children affected by PSB (15). A recent qualitative study supported this, finding that many of the community agencies involved in addressing PSB in youth lack coordinated policies or a standardized response, resulting in a fragmented approach and limited ability to identify youth affected by PSB (17).

The challenges to effectively address PSB suggest that there is an increased role for medical providers to play in the community response to this issue. Medical providers, including pediatricians, mental health providers and child abuse specialists, are in an optimal position to provide education and resources to families, including education on child development and normative sexual behaviors, and referrals for therapeutic intervention (2). Problematic Sexualized Behavior- Cognitive Behavioral Therapy (PSB-CBT) is one such intervention that has demonstrated success with this patient population, with evidence of a ten-year recidivism rate of 2% for children under 12 years of age (16, 17). Despite this role for medical providers, there is limited research available to describe the medical evaluation of children affected by PSB. The available literature on PSB is concentrated mental health evaluation and treatment and the community response to these children.

This single-center retrospective case review aims to report descriptive data on child demographics and case characteristics for children presenting to one Child Protection Program (CPP) medical specialty team for concern of PSB.

## Methods

This study was conducted at an urban, hospital-based Child Protection Program (CPP) medical specialty team in Massachusetts, United States. Children are referred to the CPP through a variety of sources when there is concern for child maltreatment, including medical professionals (emergency department providers, primary care providers, hospitalists), mental health providers, the Department of Children and Family (DCF), the District Attorney’s office, and directly by caregivers. All referrals received by the CPP for medical triage are reviewed by a CPP social worker and a CPP medical provider to determine medical recommendations.

**Figure 1:**
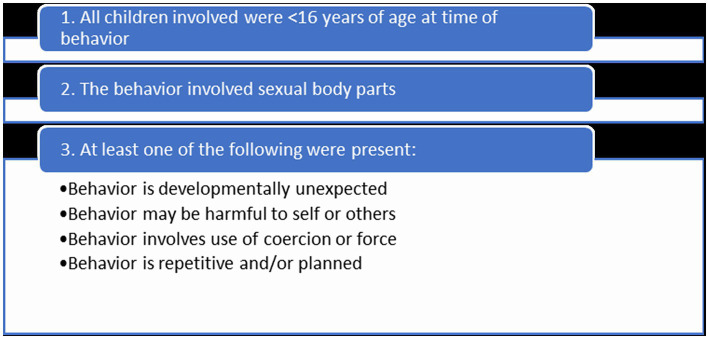
Inclusion criteria.

Chart review was conducted for all children medically triaged by CPP with concern of PSB during the period of 1/01/2020–12/31/2021. Cases were included in this analysis if they met the inclusion criteria outlined in Figure 1. Age < 16 years old was determined to be the age for inclusion in this study. While PSB is typically defined in the literature as occurring in children <12 years, the age of <16 years is used in practice in the community where this study was conducted as a threshold for how cases are assessed and addressed through resource provision.

For each index case referred to and medically triaged by CPP, data was extracted on child characteristics (age, developmental delay, and history of abuse), characteristics of the problematic sexual behavior (child’s role, types of behaviors, number of children involved, and CPP referral status of involved children) and the outcome of the medical triage. The number of children reported to be involved in the behavior with the index child but not referred to CPP for medical triage was recorded for each case as “contacts not referred”. The total number of children identified was calculated as the sum of the children referred to CPP and the contacts not referred.

The child’s role was characterized as displayer, recipient, both or unknown. The role of “Displayer” was used to identify the child exhibiting the problematic sexualized behavior, while the role of “Recipient” was used to identify the child on whom the behavior was performed. If a child both displayed and was the recipient of the behavior, their role in the behavior was designated as “Both”. This included children who were the recipient of one behavior, who then displayed a different behavior at a different point in time. The designation of “Unknown” was assigned in cases where all children involved appeared to be participating in the problematic behaviors equally without a clear initiator (for example, in sexualized play).

For each case, the outcome of the medical triage was recorded. This included whether comprehensive medical evaluation was recommended, and if this evaluation was completed by CPP or a different medical provider.

For the purpose of this study, “developmental delay” was defined as documentation of a cognitive developmental delay in the electronic medical record. A complete developmental history is routinely assessed as part of a CPP clinic medical evaluation. Visit documentation in the medical record was reviewed for children seen in the CPP clinic to determine if the child had previously been diagnosed with cognitive delay. If the child was not seen in the CPP clinic for complete medical evaluation, a review of available medical records was conducted to assess for a documented history of a diagnosis of cognitive delay. Diagnosis of developmental delay was marked as unknown if the child did not have a complete medical history with recent well child examination documented in the medical record.

The child’s history of abuse was marked as “previous abuse suspected” in situations where a history of physical or sexual abuse or neglect was disclosed at the time of initial triage or CPP clinic medical evaluation. In cases where the child was in DCF custody at time of triage, a history of abuse was presumed. History of abuse was excluded if the child received a complete medical evaluation by the CPP team and no history of abuse was identified, or if full medical record was available for review and did not include a history of abuse. All other cases were categorized as unknown.

Cases were excluded from the analysis if the problematic behavior identified was exclusively sending sexual images. While this behavior does meet the inclusion criteria used for this study, inconsistent tracking of this particular behavior over the study period would make its inclusion a source of error. Cases referred to CPP by the District Attorney’s office as part of routine forensic interview follow up were also excluded from the analysis. These cases were not routinely tracked regarding PSB concerns, and therefore, were excluded from the analysis.

Risk ratios were calculated to assess for a relationship between each of the hypothesized risk factors (history of abuse, developmental delay) and the outcome of displaying PSB. Cases with unknown history of abuse and unknown history of developmental delay were excluded from the calculation of risk ratios. Chi-square test for independence was calculated using 2 degrees of freedom and 5% level of significance for the exposures of history of abuse, no history of abuse and unknown history of abuse and the outcomes of displaying PSB and being the recipient of PSB. The outcomes of “both” displaying and being the recipient of PSB and “unknown” role in the PSB behavior were excluded from the chi-square analysis due to having fewer than 5 observations per cell.

This study was determined by the Institutional Review Board to not include human subject research. It was therefore not subject to Institutional Review Board approval.

## Results

A total of 110 index children were referred to the CPP for medical triage for a presenting concern of PSB. Through the medical triage process, an additional 114 children were identified as contacts of the index children. These contacts engaged in the PSB but were not referred to CPP for medical triage themselves. 51% of the 224 children in total who were identified as engaging in PSB, including both index children and contacts, were therefore not referred for medical specialty care.

Medical evaluation was recommended as the outcome of the medical triage for 81 (73.6%) of the 110 children referred to the CPP, but only 44 (40%) were medically evaluated by the CPP specialty team. An additional 22% received medical evaluation elsewhere, such as at their PCP. 10% of children were recommended to receive medical evaluation but were not evaluated by any medical provider (see Table 1).

**Table 1:** Children identified as being involved in problematic sexualized behavior.

	*n*	%
Total children identified	224	
Referred to CPP	110	49.1
Displayer	22	20.0
Recipient	76	69.1
Both	5	4.5
Unknown	7	6.4
Contacts not referred	114	50.9
Medical follow up of children referred to CPP	110	
Medical evaluation recommended	81	73.6
Medical evaluation completed by external provider	25	22.7
Medical evaluation completed by CPP	44	40.0

Only 22 (20%) of the 110 children referred to the CPP were the displayer of the behavior, compared to 76 (69%) the recipients of the behavior (see Table 1). Of the recipient cases, the child displaying PSB was also referred to the CPP for medical evaluation 14.5% of the time.

Characteristics of children identified as having been affected by PSB are shown in Table 2. The mean age was 1.2 years older for children displaying PSB compared to the recipients of PSB. A presumed history of abuse was found in 45.5% of children identified as displaying PSB, compared to only 25% of children identified as the recipient of PSB.

**Table 2:** Characteristics of children displaying and receiving problematic sexualized behavior.

	Displayer (*n* = 22)	Recipient (*n* = 76)	Both (*n* = 5)	Unknown (*n* = 7)
Mean age (yrs.)	8.8	7.5	6.0	10.3
Age Max	16.0	15.0	8.0	15.0
Age Min	3.0	1.5	3.0	8.0
Developmental delay (%)				
Yes	13.6%	11.8%	0.0%	28.6%
No	68.2%	64.5%	80.0%	57.1%
Unknown	18.2%	23.7%	20.0%	14.3%
History of abuse (%)				
Previous abuse suspected	45.5%	25.0%	20.0%	57.1%
No known history of abuse	27.3%	44.7%	60.0%	0.0%
History of abuse unknown	27.3%	30.3%	20.0%	42.9%
Ave. # Children involved but not referred	0.6	1.2	1.4	0.4
Displayers referred (%)				
Yes	N/A	14.5%	20.0%	57.1%
No	N/A	85.5%	80.0%	42.9%
Ave. # recipients per displayer	1.3	N/A	N/A	N/A

There was no statistically significant association identified between history of abuse or developmental delay and having displayed PSB. Chi-square test for independence found the outcomes of displaying or being the recipient of PSB to be independent from history of abuse.

## Discussion

These results highlight that the number of children who seek medical evaluation for concern of PSB is a gross underestimation of the overall prevalence of PSB in the community, thus highlighting a significant gap in identification and service delivery for these children. Less than half of the children identified as being affected by PSB were referred to CPP. Though the majority of these identified cases were recommended to receive medical evaluation, less than half of these children were medically evaluated by CPP.

The results demonstrate that children displaying PSB represent a particular area of missed opportunity for assessment, education, and intervention. Displayers are disproportionately left out of care, despite having the most potential benefit through targeted therapeutic interventions such as Problematic Sexualized Behavior- Cognitive Behavioral Therapy (PSB-CBT) (3).

These results support the existing literature in suggesting that a history of abuse is a likely risk factor for displaying PSB, with a presumed history of abuse in nearly half of the identified displayers of PSB (2, 8, 9). Future research is needed to further characterize risk factors for both displaying and being the recipient of PSB, including delineating which types of previous exposure to child abuse and/or violence most strongly correlates with increased risk.

### Limitations

Several limitations are inherent to the study’s design as a retrospective case review. There is a high percentage of cases with unknown variables, such as unknown history of abuse, which may be significantly reduced in future research with data collected prospectively. Misclassification bias is possible due to this missing documentation as well as inaccuracies within medical records. This bias may affect the identification of cases, as well as the identification of several of the risk factors for PSB discussed (i.e., developmental delay, history of abuse).

Another limitation of this study is the need to exclude certain subsets of cases due to inconsistent tracking throughout the duration of the study period. One such subset of cases includes those referred to CPP by the District Attorney’s office as part of routine forensic interview follow up. These cases were not routinely tracked regarding PSB concerns, and therefore, were excluded from the analysis. As mentioned previously, another subset of cases excluded from analysis was cases where the problematic behavior identified was the sending of sexual images. CPP’s process for managing these cases changed within the study period, resulting in inconsistent and variable tracking of these cases.

## Conclusion

The inconsistency within and across communities in how cases of PSB are addressed by child-serving professionals is likely contributing to the high percentage of children not referred for medical evaluation. Systems level changes in how children impacted by PSB are identified and referred for services across community agencies, as well as the development of a national standard of care, is needed to address these missed opportunities. This will require a coordinated, multidisciplinary response involving child welfare agencies and investigatory agencies, as well as medical and mental health providers.

## Data availability statement

The data analyzed in this study is subject to the following licenses/restrictions: protected information. Requests to access these datasets should be directed to kara.thompson@UMASSMEMORIAL.ORG.

## Ethics statement

Ethical approval was not required for the study involving humans in accordance with the local legislation and institutional requirements. Written informed consent to participate in this study was not required from the participants or the participants’ legal guardians/next of kin in accordance with the national legislation and the institutional requirements.

## Author contributions

KT: Conceptualization, Data curation, Formal Analysis, Investigation, Methodology, Writing – original draft, Writing – review & editing. SS: Conceptualization, Supervision, Writing – review & editing.
